# LCA of Barley Production: A Case Study from Cyprus

**DOI:** 10.3390/ijerph20032417

**Published:** 2023-01-29

**Authors:** Marinos Stylianou, Iliana Papamichael, Irene Voukkali, Michail Tsangas, Michalis Omirou, Ioannis M. Ioannides, Antonis A. Zorpas

**Affiliations:** 1 Laboratory of Chemical Engineering and Engineering Sustainability, Faculty of Pure and Applied Sciences, Open University of Cyprus, Giannou Kranidioti 33, Latsia, Nicosia 2220, Cyprus; 2 Department of Agrobiotechnology, Agricultural Research Institute, Nicosia 1516, Cyprus

**Keywords:** LCIA, environmental impact, agriculture, carbon footprint, global warming potential

## Abstract

Greenhouse gas emissions (i.e., carbon dioxide, methane, nitrous oxide) produced by agriculture contribute to global warming and climate change. Various practices followed by farmers in different environmental conditions contribute to the increase in the phenomena, and there is a need for immediate measures. The current study examines the environmental impact of barley production under rain-fed conditions in Cyprus. For this, four different nutrient management scenarios were investigated in order to evaluate the environmental performance of crop production, namely: (1) Nitrogen (20%), Phosphorous (20%), Potassium (10%); (2) Nitrogen (20%), Phosphorous (20%), Potassium (10%) and manure; (3) Nitrogen (25%), Phosphorous (10%), Potassium (0%); and (4) Nitrogen (25%), Phosphorous (10%), Potassium (0%) and manure. Data were collected from two different areas of Cyprus (Nicosia and Larnaca) through on-site visits and questionnaires. Life Cycle Assessment (LCA) was used as a method to quantify environmental impacts which were categorized into six impact categories: (i) acidification potential (AP), (ii) eutrophication potential (EP), (iii) global warming potential (GWP), (iv) ozone depletion potential (ODP), (v) photochemical, ozone creation potential (POCP), and (vi) terrestrial ecotoxicity (TAETP). LCA was used with system boundaries from field to harvest and a functional unit (FU) of one bale of hay. Research results showed that the addition of manure increased values in all impact categories. Comparing scenarios without manure (1 and 3) and with manure (2 and 4), the main process which contributed to GWP was field preparation, which resulted in 3 t CO_2_-Eq∙FU^−1^ and 46.96 t CO_2_-Eq∙FU^−1^, respectively. Furthermore, the highest contribution of sub-processes to GWP (kg CO_2_-Eq∙FU^−1^) was machinery maintenance (scenarios 2 and 4). The potential to reduce environmental impacts from barley and moreover, to mitigate the footprint of the agriculture sector in Cyprus is proposed by changing existing practices such as decreasing fuel consumption by agricultural machinery, and monitoring fertilizing and seeding. Conclusively, the carbon footprint of barley can be decreased through the improvement of nutrient management and cropping practices.

## 1. Introduction

In recent years, anthropogenic activities and climate change have exhibited significant effects on natural and agricultural ecosystems. For example, prolonged drought periods, floods, extreme events accompanied with a substantial increase in temperature, pests and disease outbreaks, and invasive weeds and microbes could all cause a gradual degradation of soil quality, an increase in greenhouse gas (GHG) emissions, and reduction in productivity [1,2,3]. These phenomena are more intense in semi-arid and arid regions and are particularly evident in the Eastern Mediterranean region [4,5].

The primary sector is both affected by and simultaneously contributes to climate change [6]. Growing seasons, yields, flowering and harvesting of specific crops are affected by water availability, high air temperature, soil quality, etc. In areas such as southern Europe where extreme heat events are increasing, climate change can also affect the proliferation and spread of insects, invasive weeds or diseases, which can affect future practices of croplands [1]. To overcome the challenges of climate change, the integration of organic amendments in agricultural ecosystems through alternative nutrient management schemes constitutes an important management tool. The use of organic amendments increases organic matter and nutrient availability, and enhances soil microbial activity. However, the prolonged use of such materials may have negative environmental effects on both the soil ecosystem and the crops. Towards this, the use of organic or chemical nutrient inputs should be chosen based on their ability to provide adequate nutrient amounts for the cultivated crop while having a low environmental impact [7,8]. Previous research has suggested that using such materials may increase gas emissions such as N_2_O, NH_3_ and CO_2_ due to an increase in substrate availability (C and N), and microbial activity [9,10]. The C/N ratio is the primary quality parameter that influences not only organic N mineralization and immobilization, but also N_2_O fluxes. In particular, incorporation of organic materials into soil with a low C/N ratio increases N_2_O emissions significantly, possibly due to an increase in nitrogen available forms [4,5,11]. Nitrogen is a necessary component of amino acids, which are the building blocks of plants and grain proteins, and is the nutrient which is tightly associated with increased crop production [12]. As a result, nitrogen losses from misuse in agriculture are increased, thereby posing a significant threat to the environment. There is an urgent need to develop new strategies for nitrogen management and apply new practices to cereal cultivation which will mitigate environmental impacts [13,14].

Cyprus is an island located in the eastern Mediterranean Sea and a member of the European Union since 2004. Cereal cultivation in Cyprus occupies 25% of the arable area and 43% of the total crop area, and barley is the dominant crop [15,16]. Barley is mainly cultivated in rain-fed agricultural systems exhibiting low soil fertility, while their productivity is highly dependent on the frequency and amount of annual rainfall [11]. Indeed, a 90% decrease in cereal production was observed during 1988 to 2008, and this reduction was attributed to low water availability [15]. The main use of barley in Cyprus is for feed production. The species grows under rain-fed conditions and the nutrient management strategies used usually include basal fertilization with composite fertilizers or animal manure. The fertilization in semi-arid and arid regions seems to be critical since any improvement in the nitrogen status of the crop is accompanied by an increase in water use efficiency and the productivity of the system. Thereby any changes to nutrient management could positively or negatively affect the performance of the crop. The productivity of cereals in Cyprus is relatively low compared to temperate regions and is highly susceptible to adverse environmental conditions while the country heavily relies on imports to meet its total annual needs [17]. In detail, in 2007 approximately 220,008 tons of barley were imported, whereas local production was only 37,750 tons of barley [18]. According to the Observatory of Economic Complexity (OEC), USD 28 million (M) worth of barley imports were reported, which sets Cyprus as the 33rd largest importer of barley in the world. Barley is imported to Cyprus from Romania (USD 11.5 M), Ukraine (USD 6.9 M), Italy (USD 3.16 M), Greece (USD 1.54 M), and France (USD 1.4 M) [19]. In addition, several climatic scenarios are projecting lower yields in the region mainly due to higher temperature and low precipitation [20]. These findings urge the development of new practices and adaptation of previous strategies to sustain system productivity.

**Figure 1:**
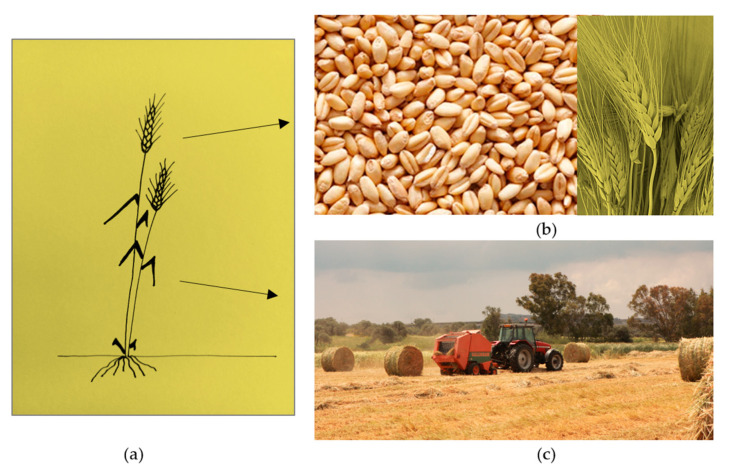
(**a**) Barley plant, (**b**) Seeds after harvesting, (**c**) Production of bale by a tractor. Photos taken by the authors.

Several studies have shown that N fertilization increases barley yield in the Mediterranean environment even under low-yielding conditions [21]. Lack of N could reduce the water use efficiency in dry regions leading to a further decrease in yield of the crop. These findings suggest that the application of organic or chemical N fertilizers will improve barley yield under dry conditions [21], however, the environmental performance of the crop is unknown when organic and chemical fertilizers are used in barley fields in semi-arid regions. The goal of the producer is the maximization of yield, which depends on the type of grain, soil type and quality, the Nitrogen Phosphorus Potassium availability (NPK), precipitation, weed management, and the proper time/conditions of harvest [22]. The production and maximization of barley bales per hectare is a critical target for all farmers especially in these years of economic crisis (Figure 1). As mentioned before, productivity depends on parameters which a farmer cannot predict such as precipitation, which in rain-fed agricultural systems is one of the crucial factors for productivity. In relation to this, farmers follow some specific practices which have different environmental footprints such as changing grain variety, increase in fertilizer input, change in harvesting period, use of different, old or new equipment, etc. [16,22].

In order to keep track of the environmental burdens of each of the different parameters affecting agricultural systems, adequate monitoring and quantification of environmental implications is of utmost importance. In this regard, Life Cycle Assessment (LCA) can be used [23,24,25,26,27]. Throughout the life cycle of a product, from the acquisition of raw materials to production, use, end-of-life treatment, recycling, and final disposal (i.e., cradle-to-grave), LCA is a standard procedure that addresses the environmental aspects and potential environmental impacts of processes or services (i.e., use of resources, carbon footprint, eutrophication, etc.) [26,27]. Over the past thirty years, it has rapidly advanced from a basic energy analysis to include a full life cycle impact assessment, life cycle costing, and social-LCA, and most recently to a more thorough life cycle sustainability analysis, which broadens the scope of conventional environmental evaluation [24,28]. It is directly applicable to the creation and enhancement of products, strategic planning, the assessment of environmental performance, the formulation of public policy, and other activities. LCA has been used in the past for the improvement of the environmental performance of specific production processes and services [23,24,25], the monitoring and assessment of CO_2_ emissions [26], the estimation of the impact of climate change on energy crops [27], as a strategic development plan in agricultural areas [28], and for a circular economy [29]. 

The goal of the current research is the investigation of the environmental impact of barley production in Cyprus through LCA. The main objective is the assessment of the environmental impact of barley under rain-fed conditions when different nutrient management scenarios are implemented to evaluate the environmental impact of the crop on specific impact categories including: (i) acidification potential (AP), (ii) eutrophication potential (EP), (iii) global warming potential (GWP), (iv) ozone depletion potential (ODP), (v) photochemical, ozone creation potential (POCP), and (vi) terrestrial ecotoxicity (TAETP). 

## 2. Methodology

### 2.1. Method Description

According to ISO 14040:2006, the LCA study should go through four phases [30,31]. In the goal and scope definition phase, the boundaries of the examined system, the functional unit (FU), and the level of the LCA’s detail are specified. In order to accomplish the objectives of the particular study, the life cycle inventory (LCI) analysis phase entails the essential input/output of data with regard to the examined system. Furthermore, during the life cycle interpretation phase, the results of the inventory and impact assessment phase are summarized and discussed, and conclusions and recommendations are formed in accordance with the goal and scope. During the interpretation, information from the life cycle impact assessment phase (LCIA) results is gathered in order for the impacts to be assessed. 

### 2.2. Area Description

**Figure 2:**
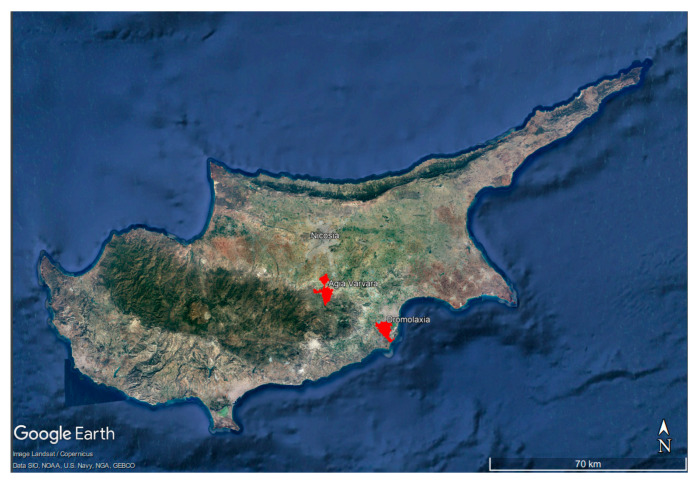
Google Earth image of the areas from which data were collected in Cyprus.

Cyprus is an island located in the eastern Mediterranean Sea and a member of the European Union since 2004. Its climate is characterized by warm and dry summers with an average annual temperature of 17.5 °C and with an increasing trend of 1.4 °C (in the case of Nicosia) [15]. Furthermore, a recent study has predicted that for the period 2031–2060, significant seasonal increases in both the mean maximum and minimum temperatures will occur [32,33]. In regard to precipitation, the wet season lasts from November to March while the driest period is from July to September. Furthermore, the amount of precipitation depends on year and location, and it has decreased by about of 100 mm on average over the last 85 years [3,15]. In parallel to the above, 9.68% of Cyprus is at environmental risk concerning land degradation potential [34]. The data constituted an average of four pilot sites, located in Nicosia (Agia Varvara) and Larnaca district (Dromolaxia) (Figure 2). Regarding soil characteristics, soil fertility and soil analysis, barley soils in Cyprus have an average PH of 8.1 and a total N content of 0.12% in dry soil. The mean precipitation of the country for the years 2018 to 2021 were as follows: 2018–2019: 785 mm; 2019–2020: 626 mm; and 2020–2021: 348 mm [35,36]. For the years 2020–2021, even if precipitation was lower, the months of rain were fit to the field cultivation schedule. Therefore, there were no differences in production from this regard.

### 2.3. Goal and Scope

**Figure 3:**
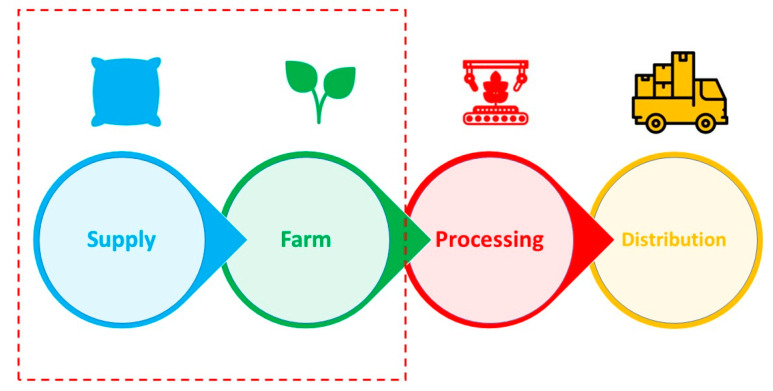
Processes of barley production. The LCA investigated the environmental impact of the farming process.

The goal of the current study was to investigate and analyze the environmental impact of barley production in typical arable land of Cyprus. The secondary objective of the current study was the investigation of the environmental effect of using different fertilizers in combination with manure. In order to fulfil a gate-to-gate approach from the supply of raw materials, field preparation and cultivation, seeding and fertilization, pest and weed management through to harvest, the LCA approach was established for the cultivation of barley production (Figure 3). Within a crop rotation, the gate-to-gate system included inputs, outputs, and field practices needed to generate 1 bale of hay of barley crop. The crops were assessed over a year, with the underlying assumption that the annual precipitation was adequate for the entire harvest cycle. While crop patterns with durations ranging from two to five years were assigned using questionnaires and production statistics from farmers, agricultural management data statistics from experimental repetitions were averaged per process.

The use of inorganic fertilizers or organic manure alone cannot sustain production [37]. Long term use of manure and fertilizer combinations results in large accumulation of nitrogen (both organic and inorganic) in soils, leading to adverse environmental effects including eutrophication and soil toxicity [38]. Still, the combination of the two is preferred by producers as it can increase carbon fractions and promote mineralization of nitrogen ions thus enhancing soil productivity in barley or wheat cropping areas [39,40]. In order to investigate the environmental impact of the combination of fertilizers and manure, four scenarios were established using the two most commonly used Nitrogen Phosphorus Potassium fertilizers (NPK) on the island:

1. Scenario 1: Production of barley in a year with fertilizer N (20%) P (20%) K (10%)—NPK 20-20-10;
2. Scenario 2: Production of barley in a year with fertilizer N (20%) P (20%) K (10%)—NPK 20-20-10 and manure;
3. Scenario 3: Production of barley in a year with fertilizer N (25%) P (10%) K (0%)—NPK 25-10-0;
4. Scenario 4: Production of barley in a year with fertilizer N (25%) P (10%) K (0%)—NPK 25-10-0 and manure.

### 2.4. Functional Unit 

The functional unit (FU) reflects a marketable product measured and explicitly specified to allow for mathematical normalization as well as for simpler comparison and measurement [31]. According to many researchers, the FU for barley production is 1 kg yield of crop product [41,42,43] or 1 hectare used [44,45,46]. However, the FU used in the current LCA for the data collection and inventory formulation was 1 bale of hay. This FU has been used in the past with satisfactory results [47]. All other products (barley grain yield) were considered as co-products of production as bales of hay are the primary measurement of feed production and the main component of barley crops. The primary inputs were agricultural chemicals (fertilizer, pesticides, etc.), energy/fuel use and seeds. The unit processes related to pre-processing of chemicals and seeds, transportation to the field, and post-harvesting impacts (such as storage, drying, etc.) were excluded.

### 2.5. Software

OpenLCA (GreenDelta, Berlin, Germany), a free and open source software created by GreenDelta, was used to conduct the LCA assessments [48]. The software allows for the importation of numerous LCA databases and LCIA methods, both free and paid for, giving the user the ability to create a life cycle system by connecting all LCI parts and to quantify the LCIA in accordance with the method employed [28].

### 2.6. Data Collection

Primary data was gathered through on-site/field inspections and a voluntary survey of planters and farmers (taking into consideration any ethical standards). This strategy aimed to strengthen the validity of LCA and generate findings in accordance with the regional agricultural and economic conditions [28].

As a result, key site-specific data were collected using a questionnaire that included questions about production, usage of chemical fertilizers and herbicides, energy, materials, equipment, fuel consumption, byproducts, and waste disposal practices. When possible, the direct emissions primarily to soil, water, and air were calculated using the primary data obtained. Furthermore, the secondary data (background data) were obtained by LCA databases (Εcoinvent, Agribalyse, etc.) and literature. Tertiary data were calculated using principal engineering concepts (mass and energy balance systems). 

### 2.7. Impact Categories

Six environmental impact categories were calculated in the LCIA including: (i) acidification potential (AP) in kg SO_2_-eq·FU^−1^, (ii) eutrophication potential (EP) measured in kg PO_4_-eq·FU^−1^, (iii) global warming potential (100 years) (GWP) measured in kg CO_2_- Eq∙FU^−1^, (iv) ozone depletion potential (ODP) in kg CFC_-11_-eq·FU^−1^, (v) photochemical, ozone creation potential (POCP) in kg C_2_H_4_-eq·FU^−1^, and (vi) terrestrial ecotoxicity (TAETP) measured in kg 1,4-DCB-Eq∙FU^−1^. These impacts are the main environmental burdens of agricultural activities and production, including but not limited to air, soil and water emissions, and have been reported elsewhere. These impacts have been used in the past by many researchers in order for the LCA analysis as depicted in Table 1. Thus, the authors chose to investigate impact categories which can be somewhat comparable with previous studies to increase novelty and reliability of results. 

## 3. Results and Discussion

### 3.1. System Modeling

**Figure 4:**
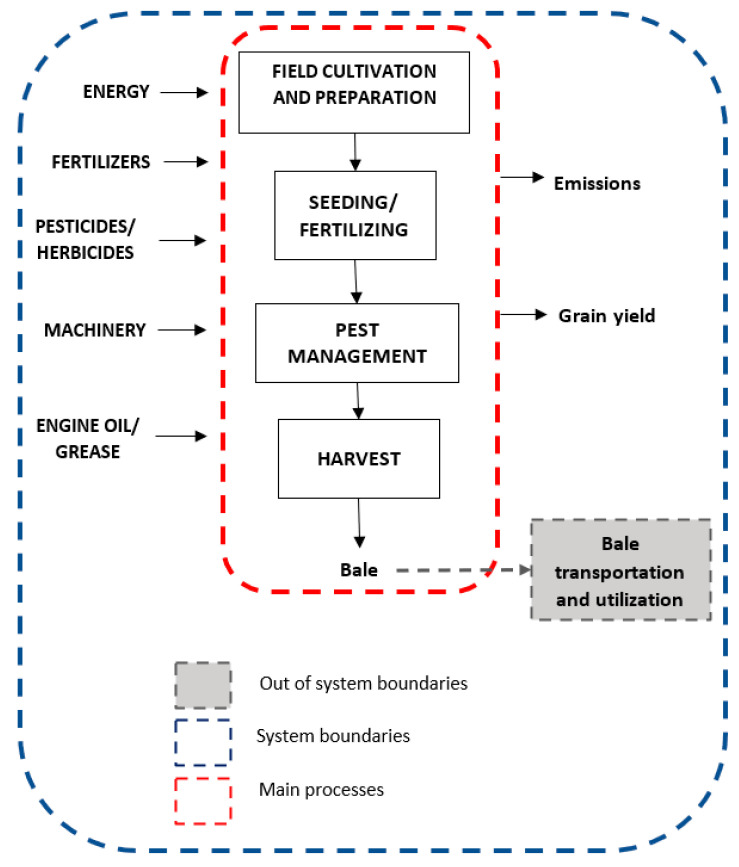
Analytical system modeling of barley production system under study.

**Figure 5:**
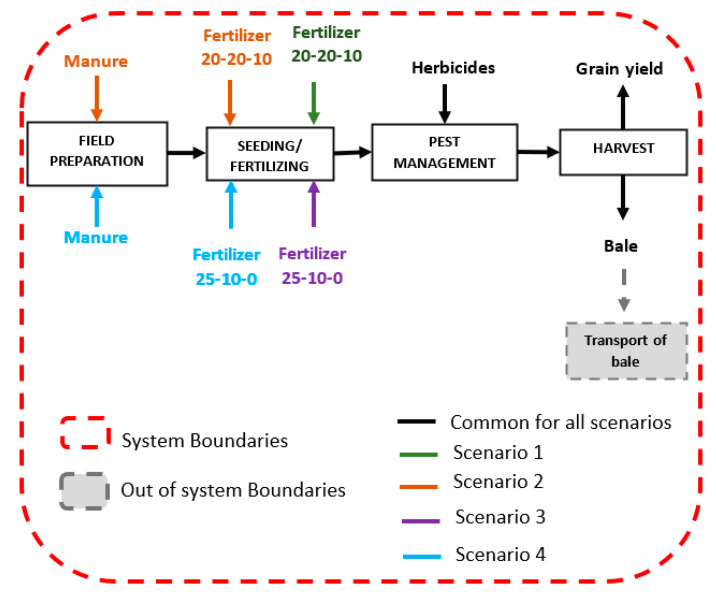
System boundaries along with the four scenarios investigated.

LCA system modeling can vary from each system under study according to the assumptions made as well as the boundaries investigated. For the environmental impact assessment of barley, Figure 4 illustrates the common inputs and outputs of the system while Figure 5 depicts the system boundaries as well as the flows used according to the different scenarios investigated. The “emissions” mentioned in Figure 4 include all emissions investigated (i.e., water, soil, air, etc.). 

### 3.2. Life Cycle Inventory (LCI) Analysis

The input and output flows of the system were analyzed in LCI. Questionnaires and personal communications were used to collect the necessary primary data for the pilot areas as presented in Table 2. For secondary data acquisition, data sources from existing databases (i.e., Agribalyse, Eco-invent) and scientific literature of similar previous studies were used. Lastly, tertiary data were calculated using basic engineering calculations.

Regarding the specifications of equipment and materials used, the sites investigated used the same or similar branding. Where outliers were in place, the authors used the majority of answers in order to provide an equal basis for results presented. Specifically the equipment presented in Table 2 are specified as follows: (i) Harrow (UNIA Cost Premium 3.5 m, Grudziadz, Poland), (ii) Lorry (Mercedes, 6.5 m, Stuttgart, Germany), (iii) Pneumatic seeder (UNIA Amber, 4 m, Poland), (iv) Baler (McHale, V6-750, Ballinrobe, Ireland), (v) Harvester (Claas Tucano 320, Vercelli, Italy), (vi) Tractor (John Deere, 190 hp, Moline, IL, USA). Where data were not available on the OpenLCA databases, the authors used assumptions as described in Section 3.3. 

### 3.3. Data Quality

For the collection and assessment of primary data, questionnaires and surveys were used while secondary data were obtained using trustworthy LCA databases. Data quality assessment is depicted in Table 3 for primary and secondary data, including average results from all four scenarios prepared according to guidelines by Ecoinvent v3.3 [54]. The five quality indicators used were: reliability, completeness, temporal correlation, geographical correlation and further technological correlation, scaled from 1 to 5 (1 being the best correlation and quality of data). Primary data constituted high quality inputs while secondary data arose from databases and concerning background systems could be substantially improved. One of the main areas lacking data quality is the fact that even if Mediterranean areas are included—for the most part—in the databases, there were no available data for Cyprus and thus the authors had to choose data concerning areas with similar climatic conditions (i.e., Spain). Additionally, there were limitations in the actual emissions from fertilizer use, as data for the NPK 20-20-10 and NPK 25-10-0 commonly used in Cyprus are not available on LCA databases. Authors had to use basic engineering and chemistry calculations in order to provide adequate conversions according to the data available. Further assumptions included the origination of barley seeds (which were available only for France) as well as the brand of machinery used (i.e., tractors, baler, lorry, etc.) which could not be specified from LCA databases but were chosen in close proximity with the questionnaires provided. Furthermore, packaging waste (i.e., fertilizer and pesticide waste) could not be directly imported as the brand of fertilizer and pesticide used was not available. Therefore, authors used the disposal of plastic waste specific to the composition of fertilizer and pesticide packaging of the current system (i.e., waste polyethylene).

### 3.4. Life Cycle Impact Analyses

OpenLCA was used to calculate the impact categories of the LCA. CML 2001 was used for the calculation of the data and the impacts of each category are depicted in Table 4.

According to the “Farm to Fork Strategy” of the EU, a 50% decrease in nutrient loss is required without deteriorating soil fertility but also a 20% reduction in inorganic fertilizer use in order to create a fair, sustainable and environmentally friendly food system [44,55]. Simultaneously, the European Green Deal targets as a legal obligation carbon neutrality until 2050 with a gradual decrease in GHGs emissions to 55% by 2030 (compared with 1990 levels) [55,56]. The carbon footprint and GHG emissions level of agricultural practices vary widely around the member states of the EU. According to the European Environment Agency [57], there has been an estimated 2% decline in GHG emissions from agriculture until 2030 compared to 2005 levels. Croatia, Malta, Romania and Greece achieved a decrease of 10% while Estonia, Hungary, Bulgaria and Latvia increased their agricultural emissions by 10%. 

Regarding this, LCA methodology has been used by many studies in the past as an effective tool for monitoring and improving the eco-profile of agricultural processes [28,58,59,60,61]. The necessity of monitoring agricultural practices lies with the urgent need to halt the environmental burden of the agricultural sector while also obliging and keeping up with EU legislations and strategies. According to Statista [62], agriculture is responsible for 1.77% of Cyprus’ GDP, while the three main components of arable land are cereals, fodder crops, and fallow land accounting for 33,280 ha (28%), 32,860 ha (28%), and 9470 ha (8%), respectively in 2010 [63]. Simultaneously, the yearly GHG emissions of the agricultural sector in Cyprus amounted to 0.55 tons of CO_2_-Eq in 2020, indicating a 5.77% increase [64].

Various FUs have been used in the past to study the life cycle of barley production for the six impact categories that were investigated: AP, EP, GWP, ODP, POCP, and TAETP [13,27,47,53,65]. While literature-extracted LCA systems may be useful for some findings, they cannot yet supply all the knowledge required to move toward farms with zero waste and carbon footprints. At the same time, the differentiation between the use of fertilizers alone and the impact of manure and fertilizer use on the impact categories has not been investigated on the island. 

Barley is one of the most important crops in Cyprus, yet, to the authors’ knowledge, very few studies have been performed on this crop [27]. Using the FU of one bale of hay is very convenient as, according to the agricultural sector investigation, most farmers use this unit of measurement for their crop production. Therefore, the results indicated could provide a better understanding on the environmental implications of barley production in Cyprus. 

When investigating the values of the impact categories for one bale of hay in Cyprus within the existing literature, the data are comparable for GWP, TAETP and EP while AP and ODP show significant differentiations. Considering GWP as the most important impact category due to the need for GHGs and carbon footprint monitoring, Lechon et al. [49] indicated 136 t CO_2_-Eq/ha, amounting to approximately 23,100 kg CO_2_-Eq∙FU^−1^ (FU: one bale produced). Still, the difference in AP and ODP compared to the existing literature constitutes an area of interest where further investigation is necessary. At the same time, the addition of manure in Scenarios 2 and 4 seems to increase the value of all impact categories (Table 4). According to the system model, this is due to the use of extensive machinery (i.e., lorries) to transport and spread manure for field cultivation. This can be seen for TAETP values as, despite the decrease in fertilizer inputs (NPK 20-20-10 and 25-10-0) for each of the two scenarios, TAETP values increased for scenarios 2 and 4 by 38.26 kg 1,4-DCB-Eq∙FU^−1^. This could imply that the manure: fertilizer ratio used in the agricultural cultivation of barley does not significantly change soil toxicity but rather machinery practices and maintenance have a higher impact on environmental burdens. 

**Figure 6:**
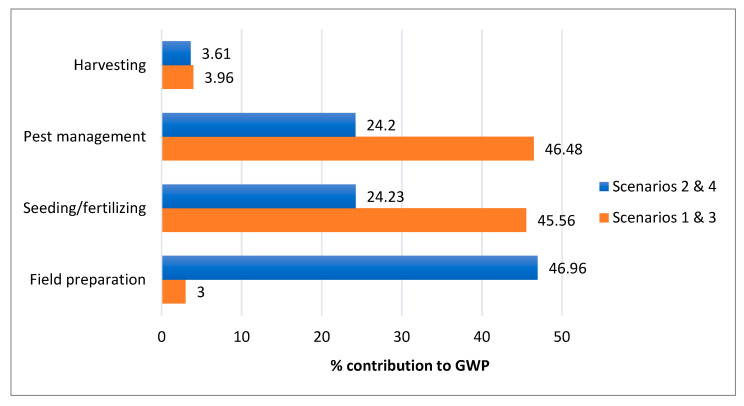
Percentage contribution of scenarios 1 and 3, and 2 and 4 to GWP values per process according to the results of the OpenLCA.

**Figure 7:**
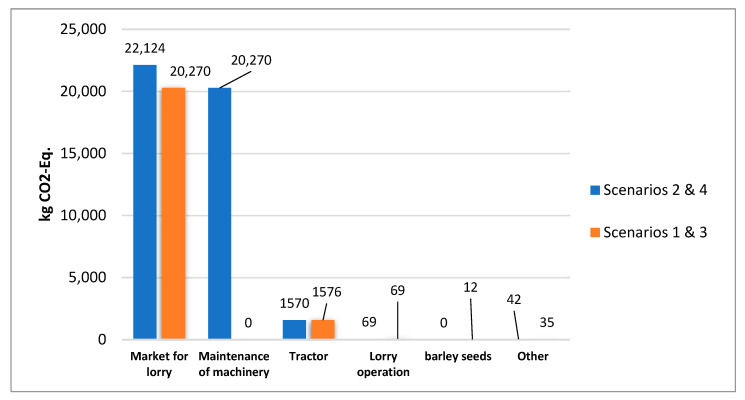
Six highest impact processes according to the results of the OpenLCA in kg CO_2_-Eq∙FU^−1^ for the measurement of GWP.

Verdi et al. (2022) [44] investigated the differences between the organic and conventional farming of wheat using LCA. Their results indicated that indeed, the most relevant contribution was fuel consumption from agricultural machinery, while ploughing was shown to produce a 29–32% impact on the total fuel consumption. However, the contribution of machinery in agricultural activity shows strong fluctuations depending on climate conditions, production system (i.e., organic), crop yield, etc. In the current study, seeding and fertilizing process, pesticide use and maintenance of machinery (i.e., lorries) seems to have the largest contributions to impact category values in all four scenarios (Figure 6 and Figure 7). For GWP, maintenance of machinery had a 47% contribution in Scenarios 2 and 4, amounting to 20,270 kg CO_2_-Eq∙FU^−1^ (Figure 6 and Figure 7) when the addition of manure took place. The four scenarios showed little difference when it came to harvesting. 

The implementation of new methodologies to calculate the carbon footprint of different crops is of utmost importance in order to coordinate economic development and monitor and mitigate environmental burdens, while at the same time remain in line with EU legislations and strategies for the farming system. Therefore, the monitoring of the carbon footprint and GWP of agricultural processes was one of the main impact categories of major interest [66]. Carbon footprint can be defined as carbon emissions induced by a process or production in a growing season. To this end, Zhang et al. (2017) [66], calculated the carbon footprint of three major crops. According to their results, wheat production produced 5455 kg CO_2_ eq/ha. Kashyap and Agarwal (2021) [67] investigated rice and wheat production in Punjab, where the carbon footprint of rice and wheat production per unit area were 8.80 and 4.18 CO_2_ eq/ha, respectively, arising from residue burning, fertilizer use and direct methane emissions. The authors also indicated that larger farms seemed to have lower carbon footprints per ton of crop produced than smaller farms; e.g., 39% lower CF per ton of rice compared to small farms [67].

In addition, according to Masuda (2016) [58], nitrogen fertilizers affect both yield and eco-efficiency in barley or wheat cultivation and production. There are a number of efficient methods for scaling back nitrogen fertilizer use in order to achieve a net reduction in nitrogen input while maintaining wheat yields. For instance, soil analyses, such as a NO_3_ test performed before planting, can help prevent excessive nitrogen fertilizer application. Indeed, incorporation of nitrogen fertilizer into the soil and side-dress application reduces N losses such NH_3_ volatilization and increases nitrogen use efficiency of the crop. Moreover, nitrogen fertilizer use is decreased as a result of smart agricultural practices which are based on satellite navigation and auto-guidance systems since there is less overlap in fertilizer application [58].

According to the literature [68,69,70], 1–5% of N distributed during fertilization is lost as nitrogen oxide contributing greatly to global warming and soil toxicity. Therefore, the impact of fertilization is a serious issue for farming. Fallahpour et al. (2012) [51] reported a global warming mitigation potential from organic fertilizers of 80% compared to conventional chemical fertilizers. Thus, precision in agricultural practices is not only a matter of increasing product value but also mitigating soil deterioration that would, in time, decrease agricultural activities and, as a result, economic inflow. Still, there seem to be limitations caused by technological barriers in less developed agricultural systems. For instance, the use of slow-release N-fertilizers and nitrification inhibitors to aid in long term sustainability are not yet affordable for small farms and individual producers [44].

Different technologies can be implemented to monitor the carbon footprint of agricultural ecosystems. Carbon labelling has been used for informing consumers about the GHG emissions over the entire lifetime of products, revealing a pathway towards sustainable practices and awareness in everyday life. Acting as a guide to low-carbon production strategies, informing the public about the implications of overconsumption of products according to real life data could act as a catalyst for the establishment of low-carbon products which could alter agricultural practices all the way back to field cultivation. The over ending increase in carbon emissions calls for a socioeconomic transformation around the world, urgently urging governments and local authorities towards more sustainable practices across different sectors [66].

## 4. Conclusions

In the present study, LCA was used as a method to assess the environmental impact of barley production through four scenarios based on different nutrient management practices. The results revealed that the application of NPK fertilizers in combination with manure in two separate application periods increased all environmental impact categories studied, namely AP, GWP, EP, POCP, ODP, and TAETP. Specifically, for one bale of hay, for Scenarios 2 and 4, the addition of manure appears to boost the values of all impact categories. According to the system model, this is due to the substantial machinery (i.e., lorries) used to carry and distribute manure for field cultivation. This is demonstrated by the fact that despite the decrease in fertilizer inputs (NPK 20-20-10 and 25-10-0) for each of the two scenarios, TAETP levels increased by 38.26 kg 1,4-DCB-EqFU-1 for scenarios 2 and 4. This might suggest that the manure-to-fertilizer ratio employed in barley cultivation does not considerably affect soil toxicity, but equipment operations and maintenance have a greater influence on environmental loads. For Scenarios 2 and 4, when manure was added, the maintenance of machinery had a 47% contribution to GWP, equal to 20,270 kg CO_2_-EqFU-1. Regarding harvesting, there was minimal variation between the four scenarios.

According to these results for the production of one bale of hay, the important sub-processes that contribute to GWP are: machinery maintenance, market for lorry, fertilizing and seeding and, consequently, fuel consumption from extra agricultural machinery used. The greenhouse gas emissions from the production of one bale of hay can be reduced by changing nutrient management and cropping practices and as a result mitigation of carbon footprint of barley in Cyprus can be achieved. The investigation of barley production in Cyprus is not yet elaborated in depth, although the crop constitutes one of the main products of the agricultural sector. Therefore, further investigation into the complete supply chain regarding the environmental burden of production is needed as well as the integration of other agricultural practices concerning nutrient management on a national level. 

## Figures and Tables

**Table 1:** Impact categories results of LCA analysis for barley crops from previous studies.

Impact Category	Results	FU Used	Source
AP (kg SO_2_-Eq∙FU^−1^)	54.02	1 ha	[49]
GWP (t CO_2_-Eq∙FU^−1^)	136	1 ha	[49]
	37.1–44.3	1 MJ of crop product	[27]
	270	1 t of crop product	[50]
EP (kg PO_4_-Eq∙FU^−1^)	2.358	1 ha	[49]
	3.0	1 t of grain yield	[51]
	0.0098–0.010	1 kg of starch	[52]
POCP (kg ethylene-Eq∙FU^−1^)	8.86	1 ha	[49]
ODP (kg CFC-11-Eq∙FU^−1^)	0.004	1 ha	[49]
	2.84 × 10^−8^	1 kg grain produced	[53]
	989.84 × 10^−6^ DALY *	98,700 hay bales	[47]
TAETP (kg 1,4-DCB-Eq∙FU^−1^)	0.00145	1 kg grain produced	[53]
	5539 Specied per year **	98,700 hay bales	[47]

AP = Acidification Potential, EP = Eutrophication Potential, GWP = Global Warming Potential (100 years), ODP = Ozone Depletion Potential, POCP = Photochemical Ozone Creation Potential, TAETP = Terrestrial Ecotoxicity (100 years). * DALY = Disability Adjusted Life Years. ** Species per year used as a unit for measuring the ecosystem quality, quantified in loss of species per year.

**Table 2:** Inventory data expressed in units/FU for each given scenario.

	Unit (per FU)	Scenario 1	Scenario 2	Scenario 3	Scenario 4
Harrow	h	0.027	0.027	0.027	0.027
Land use	ha/yr	0.169	0.169	0.169	0.169
Manure	t	0	5.653	0	5.653
Average mineral fertilizer, as K2O	Kg	1.699	0.906	0	0
Average mineral fertilizer, as N	kg	3.398	1.811	3.4225	2.264
Average mineral fertilizer, as P_2_O_5_	Kg	3.398	1.811	1.369	0.905
Lorry	items	2	2	2	2
Barley Seeds	kg	18.216	18.216	18.216	18.216
Pneumatic seeder	h	0.0268	0.0268	0.0268	0.0268
Emissions from herbicides	kg	641.026	641.026	641.026	641.026
Baler	ha	0.169	0.169	0.169	0.169
Diesel	L	0.0214	0.0214	0.0214	0.0214
Electricity	kWh	1.290	1.290	1.290	1.290
Harvester	h	0.0214	0.0214	0.0214	0.0214
Tractor	items	1	1	1	1
Barley grains produced	t	0.641	0.641	0.641	0.641
Fertilizer packaging waste	gr	33.979	18.111	33.979	18.111
Herbicide packaging waste	gr	0.0342	0.0342	0.0342	0.0342
Bale	items	1	1	1	1

**Table 3:** Data quality analysis.

Quality Indicator	Primary Data	Secondary Data
Reliability	2	5
Completeness	1	3
Temporal correlation	1	4
Geographical correlation	1	4
Further technological correlation	1	3

**Table 4:** Life Cycle Impact assessment for the four scenarios.

Impact Category	Scenario 1	Scenario 2	Scenario 3	Scenario 4
AP (kg SO_2_-Eq∙FU^−1^)	125	242	124	242
GWP (kg CO_2_-Eq∙FU^−1^)	24,000	46,100	24,000	46,100
EP (kg NOx-Eq∙FU^−1^)	41.93	100.7	41.92	100.7
POCP (kg ethylene-Eq∙FU^−1^)	8.86	19.21	8.86	19.21
ODP (kg CFC-11-Eq∙FU^−1^)	0.0035	0.0051	0.0035	0.0051
TAETP (kg 1,4-DCB-Eq∙FU^−1^)	8.48	46.74	8.47	46.73

AP = Acidification Potential, EP = Eutrophication Potential, GWP = Global Warming Potential (100 years), ODP = Ozone Depletion Potential, POCP = Photochemical Ozone Creation Potential, TAETP = Terrestrial Ecotoxicity (100 years).

## Data Availability

The data presented in this study are available on request from the corresponding author.
